# A Snake Venom-Secreted Phospholipase A_2_ Induces Foam Cell Formation Depending on the Activation of Factors Involved in Lipid Homeostasis

**DOI:** 10.1155/2018/2547918

**Published:** 2018-06-14

**Authors:** Elbio Leiguez, Karina Cristina Giannotti, Mariana do Nascimento Viana, Márcio Hideki Matsubara, Cristina Maria Fernandes, José Maria Gutiérrez, Bruno Lomonte, Catarina Teixeira

**Affiliations:** ^1^Pharmacology Laboratory, Butantan Institute, São Paulo, SP, Brazil; ^2^Clodomiro Picado Institute, University of Costa Rica, San José 11501, Costa Rica

## Abstract

MT-III, a snake venom GIIA sPLA_2_, which shares structural and functional features with mammalian GIIA sPLA_2_s, activates macrophage defense functions including lipid droplet (LDs) formation, organelle involved in both lipid metabolism and inflammatory processes. Macrophages (M*Φ*s) loaded with LDs, termed foam cells, characterize early blood vessel fatty-streak lesions during atherosclerosis. However, the factors involved in foam cell formation induced by a GIIA sPLA_2_ are still unknown. Here, we investigated the participation of lipid homeostasis-related factors in LD formation induced by MT-III in macrophages. We found that MT-III activated PPAR-*γ* and PPAR-*β*/*δ* and increased the protein levels of both transcription factors and CD36 in macrophages. Pharmacological interventions evidenced that PPAR-*γ*, PPAR-*β*/*δ*, and CD36 as well as the endoplasmic reticulum enzymes ACAT and DGAT are essential for LD formation. Moreover, PPAR-*β*/*δ*, but not PPAR-*γ*, is involved in MT-III-induced PLIN2 protein expression, and both PPAR-*β/δ* and PPAR-*γ* upregulated CD36 protein expression, which contributes to MT-III-induced COX-2 expression. Furthermore, production of 15-d-PGJ_2_, an activator of PPARs, induced by MT-III, was dependent on COX-1 being LDs an important platform for generation of this mediator.

## 1. Introduction

Secreted PLA_2_ (sPLA_2_) enzymes comprise the largest family of PLA_2_s and comprise 12 isoforms subdivided in structural collections I/II/V/X and two other atypical structural collections (III and XII) [1, 2]. sPLA_2_-IIA is often referred as the inflammatory sPLA_2_, but the molecular mechanism underlying its proinflammatory action is not fully understood. These enzymes are able to release fatty acids from cell membranes, leading to subsequent production of lipid mediators. Distinct and unique roles of these enzymes in diverse biological events, including digestion, host defense, reproduction, and development of inflammatory diseases and metabolic disorders, have been described [2].

sPLA_2_s are major protein components of *Bothrops* snake venoms and are classified into groups I and II, according to their biochemical and structural characteristics. Noteworthy, sPLA_2_s from *Bothrops* snake venoms share structural and functional features with mammalian inflammatory GIIA sPLA_2_s, which are found in high concentrations in inflammatory exudates [3, 4]. Our group has previously demonstrated that an Asp49 sPLA_2_, named myotoxin-III (MT-III), isolated from *Bothrops asper* snake venom, activates inflammatory functions of macrophages, including formation of lipid droplets (LDs) and upregulation of perilipin 2 (PLIN2), a scaffold protein involved in LD assembly and macrophage differentiation into foam cells [5–7]. However, the mechanisms involved in foam cell formation induced by sPLA_2_ MT-III are still unclear.

LDs are cytoplasmic organelles, present in high levels in foam cells, that are comprised by a hydrophobic core of neutral lipids (triglycerides and cholesterol esters) surrounded by a phospholipid monolayer and a growing list of associated proteins [7–9]. LDs are found in inflammatory leukocytes and described as rich deposits of esterified arachidonic acid (AA), a precursor of eicosanoids, and enzymes necessary for their synthesis, including cyclooxygenases (COX) and prostaglandin E_2_ synthase [10]. The biogenesis of LDs is a tightly regulated process in which lipid homeostasis-related transcription factors, such as peroxisome proliferator-activated receptors (PPARs), play a key role. Activated PPARs upregulate the uptake of free fatty acids by increasing the expression of the fatty acid transporter CD36 [11, 12] and LD formation through increased expression of LD-associated proteins, such as PLIN2. Upregulation of PPAR-*γ* and PPAR-*β/δ* has been shown in foam macrophages [13], and deletion of these transcription factors resulted in a decreased number of LDs in cells stimulated with pathogens or free fatty acids. However, the implication of these factors in foam cell formation induced by group IIA secreted PLA_2_s remains unknown. In addition, the endoplasmic reticulum (ER) enzymes acyl-CoA:cholesterol acyltransferase (ACAT) and diacylglycerol O-acyltransferase (DGAT), responsible for the synthesis of cholesterol (ChE) and triacylglycerol (TG), respectively, have also been associated with foam cell formation in diverse pathological and inflammatory conditions [14]. Due to the tight association between an unbalanced lipid homeostasis and foam cell formation seen in diverse inflammatory conditions, we investigated the participation of distinct lipid homeostasis-related factors, such as the transcription factors PPAR-*γ* and PPAR-*β*/*δ*, the ER enzymes DGAT and ACAT, and CD36 scavenger receptor in LD formation induced by the snake venom GIIA sPLA_2_, MT-III, in macrophages.

## 2. Materials and Methods

### 2.1. Chemicals and Reagents

Heparin was obtained from Roche Quimicos Farmaceuticos S.A. (Rio de Janeiro, S.A., Brazil) and Hema-3 stain from Biochemical Sciences (Swedesboro, NJ, USA). MTT and L-glutamine were obtained from USB Thermo Fisher Scientific (Cleveland, OH, USA). GW9662, GSK0660, and A922500 were purchased from Merck KGaA (Darmstadt, HE, Germany). TMP-153 and 15-d-PGJ2 antibody were obtained from Cayman Chemical (Ann Arbor, MI, USA). PPAR-*γ* and PPAR-*β*/*δ* antibodies were purchased from Santa Cruz Biotechnology (Dallas, TX, USA). CD36 antibody was obtained from R&D Systems (Minneapolis, MN, EUA). 15-deoxy-*Δ*^12,14^-PGJ2 ELISA kit was purchased from Enzo Life Sciences (Farmingdale, NY, USA). E-toxate LAL kit, mouse mAb anti-*β*-actin, EDAC, sulfo-N-succinimidyl oleate (SSO), and Nile Red were purchased from Sigma-Aldrich (St. Louis, MO, USA). Secondary antibodies, anti-mouse and anti-guinea pig, conjugated to HRP and nitrocellulose membrane, were obtained from GE Healthcare (Buckinghamshire, UK). Gentamicin was purchased from Schering-Plough (Whitehouse Station, NJ, USA). DMSO and BSA were obtained from Amresco (Solon, OH, USA). RPMI 1640, thiocarbohydrazide, and OsO_4_ were purchased from Sigma-Aldrich (St. Louis, MO, USA). PLIN2 antibody, AlexaFluor 488 antibody, and iodide propidium were purchased from Thermo Fisher Scientific (Sao Paulo, S.P., Brazil). GA, TG, and all salts used were obtained from Merck (Darmstadt, Germany). PFA was purchased from Electron Microscopy Sciences (Hatfield, PA, USA). Fluoromont G was purchased from Molecular Probes (Eugene, OR, USA). Donkey serum was obtained from Jackson ImmunoResearch Laboratories (West Grove, PA, USA). Triton-X was obtained from Union Carbide (Houston, TX, USA).

### 2.2. Animals

Male Swiss mice (18–20 g) were obtained from Butantan Institute (São Paulo, Brazil). Animals were housed in a temperature-controlled room (22–24°C) with a 12 h light-dark cycle and fresh water and food ad libitum. This study was approved by the Butantan Institute Animal Experimentation Ethics Committee (reference number 760/10) in accordance with the procedures laid down by the Universities Federation for Animal Welfare.

### 2.3. Phospholipase A_2_ (MT-III)

MT-III was isolated from *Bothrops asper* venom by ion-exchange chromatography on CM-Sephadex C-25 using the conditions described by Gutierrez and Lomonte [15] (verificar referência), followed by RP-HPLC on a C8 semipreparative column (10 × 250 mm; Vydac) eluted at 2.0 mL/min with a 0–70% acetonitrile gradient containing 0.1% (*v*/*v*) trifluoroacetic acid, during 30 min, on an Agilent 1200 instrument monitored at 215 nm. Homogeneity of the final preparation was assessed by analytical RP-HPLC on a C4 column (4.6 × 150 mm) using a 0–60% acetonitrile gradient. The absence of endotoxin contamination in the MT-III preparation was demonstrated by the quantitative Limulus amebocyte lysate (LAL) test [16], which revealed undetectable levels of endotoxin (<0.125 EU/mL).

### 2.4. Harvesting of Macrophages

Peritoneal macrophages were harvested from mice 4 days after i.p. injection of 1 mL of 3% thioglycolate. Animals were killed under CO_2_ atmosphere, and cells were harvested by washing peritoneal cavities with 3 mL of PBS, pH 7.2, containing 10 IU/mL heparin. Aliquots of the washes were used for total cell counts in a Neubauer chamber after dilution (1 : 20, *v*/*v*) in Turk solution (0.2% crystal violet dye in 30% acetic acid). To verify the predominance of macrophages in samples, differential cell counts were performed on smears stained with Hema3. More than 95% of the cell population consisted of macrophages, as determined by conventional morphological criteria. The remaining wash volumes were centrifuged at 500 ×g for 6 min (4°C), and the cell pellets were used for subsequent studies after suitable dilutions.

### 2.5. Stimulation and Treatment of Macrophages

Macrophages were seeded on glass coverslips at a density of 2 × 10^5^ cells/coverslip and allowed to attach for 30 min at 37°C under a 5% CO_2_ atmosphere. Nonadherent cells were removed by washing with PBS. Cell monolayers were cultured for 1 h in RPMI-1640 supplemented with 40 *μ*g/mL gentamicin sulfate and 2 mM L-glutamine at 37°C and 5% CO_2_ and were then challenged with MT-III (0.4 *μ*M) or medium (control). This concentration of MT-III was selected on the basis of the previous studies, since it induces the formation of LDs in macrophages (Leiguez et al., [17]). Where appropriate, the following inhibitors were used: GW9662 (10 *μ*M), inhibitor of PPAR-*γ*; GSK0660 (10 *μ*M), inhibitor of PPAR-*β*/*δ*; SS0 (25 *μ*M), inhibitor of CD36; A922500 (100 nM), inhibitor of DGAT enzyme; and TMP-153 (100 nM), inhibitor of ACAT enzyme. All stock solutions were prepared in DMSO and stored at −20°C. Aliquots were diluted in RPMI-1640 to the required concentration immediately before use. The final DMSO concentration was always lower than 1% and had no effect on lipid body numbers. All pharmacological inhibitors were added between 30 and 60 min before stimulation of macrophages with MT-III or medium (control). Cells treated with the inhibitors were analyzed for viability by the tetrazolium-based (MTT) colorimetric assay. No significant changes in cell viability were registered with any of the above agents or vehicle at the concentrations used (data not shown).

### 2.6. Lipid Droplet Staining and Quantification

Analysis of lipid droplet numbers was performed in osmium-stained cells. In brief, macrophages (2 × 105 cells) that adhered to glass coverslips were fixed in 4% PFA in 0.1 M phosphate buffer, pH 7.2, for 15 min and stained with OsO_4_. The coverslips were then rinsed in 0.1 M phosphate buffer, stained in 1% OsO_4_ (30 min), rinsed in deionized H_2_O, immersed in 1.0% thiocarbohydrazide (5 min), rinsed again in 0.1 M phosphate buffer, restained with 1% OsO_4_ (3 min), rinsed with H_2_O, and then dried and mounted. The morphology of the fixed cells was observed, and round osmiophilic structures were identified as lipid droplets, which were then counted under phase-contrast microscopy using the 100x objective lens in 50 consecutively scanned leukocytes in each coverslip. For assays with fluorescent-labeled lipid droplets, macrophages (2 × 10^5^ cells) that adhered to glass coverslips were incubated with Nile Red staining solution freshly prepared in 0.1 M phosphate buffer (10 *μ*g/mL) for 20 min at room temperature and washed with phosphate buffer. After several washes, the coverslips were mounted with fluoromont G and examined under a fluorescence microscope equipped with the appropriate filter (Zeiss LSM 510 Meta, Dublin, CA, USA).

### 2.7. Western Blotting

Aliquots of MT-III-stimulated and nonstimulated cells (2 × 106 cells) were lysed with 100 *μ*L of sample buffer (0.5 M Tris–HCl, pH 6.8, 20% SDS, 1% glycerol, 1 M *β*-mercaptoethanol, and 0.1% bromophenol blue) and boiled for 10 min. Samples were resolved by SDS polyacrylamide gel electrophoresis (SDS-PAGE) on 10% bis-acrylamide gels overlaid with a 5% stacking gel. Proteins were then transferred to nitrocellulose membrane (GE Healthcare, Buckinghamshire, UK) using a Mini Trans-Blot® (Bio-Rad Laboratories, Richmond, CA, USA). The membranes were blocked for 1 h with 5% nonfat dry milk in TTBS (20 mM Tris, 100 mM NaCl, and 0.5% Tween 20) and incubated with primary antibodies against PLIN2 (1 : 2000 dilution) or PPAR-*γ* or PPAR-*β*/*δ* or CD36 (1 : 2000) and *β*-actin (1 : 3000) for 1 h. They were then washed and incubated with the appropriate secondary antibody conjugated to horseradish peroxidase. Detection was made by the enhanced chemiluminescence (ECL) method according to the manufacturer's instructions (GE Healthcare, Buckinghamshire, UK). Band densities were quantified with a GS-800 Densitometer (Bio-Rad Laboratories, Richmond, CA) using the image analysis software from Molecular Analyst® (Bio-Rad Laboratories, Richmond, CA).

### 2.8. 15-d-PGJ_2_ Quantification

Release of 15-d-PGJ_2_ was determined by enzyme immunoassay using commercial kits. In brief, 50 *μ*L aliquots of each sample were incubated with the eicosanoids conjugated with acetylcholinesterase and the specific rabbit antiserum in 96-well plates were coated with anti-rabbit IgG mouse monoclonal antibody. After addition of the substrate, the absorbance of the samples was recorded at 405 nm in a microplate reader (Labsystems Multiskan), and concentrations of 15-d-PGJ_2_ were estimated from standard curves.

### 2.9. Triacylglycerol and Cholesterol Quantification

The amounts of triacylglycerol and cholesterol/cholesterol ester were measured using triacylglycerol or Cholesterol/Cholesterol Ester Detection Kits according to the manufacturer's instructions (Abcam) in cells treated with medium or MT-III.

### 2.10. Immunocytochemistry Analysis

Detection of 15-d-PGJ_2_ in MT-III-stimulated macrophages was performed by 15-d-PGJ_2_ immunostaining. Cells were fixed and permeabilized in 1% EDAC [18] in calcium- and magnesium-free Hank's balanced salt solution (HBSSˉ/ˉ). The macrophages were blocked with 0.5% normal donkey serum in 0.1 M phosphate buffer for 60 min and then washed with HBSSˉ/ˉ and incubated for 1 h with monoclonal antibodies against 15-d-PGJ_2_ (1 : 100). After further washes, the cells were incubated with goat anti-rabbit Alexa Fluor 488 (1 : 250) and Nile Red solution (1 : 250) for 1 h. The coverslips were then washed three times and mounted with Fluoromount-G containing propidium iodide (Invitrogen) and examined under a confocal laser scanning microscope (Zeiss LSM 510 Meta, Dublin, CA, USA).

### 2.11. Statistical Analysis

Data are expressed as the mean ± standard error of mean (SEM) of at least three independent experiments. Multiple comparisons among groups were performed by one-way analysis of variance (ANOVA) followed by Tukey's test. Values of probability lower than 5% (*p* < 0.05) were considered significant.

## 3. Results

### 3.1. PPAR-*γ* and PPAR-*β*/*δ* Contribute to LD Formation, but Only PPAR-*β/δ* Is Involved in Increase of PLIN2 Protein Expression in Macrophages Stimulated by MT-III

**Figure 1:**
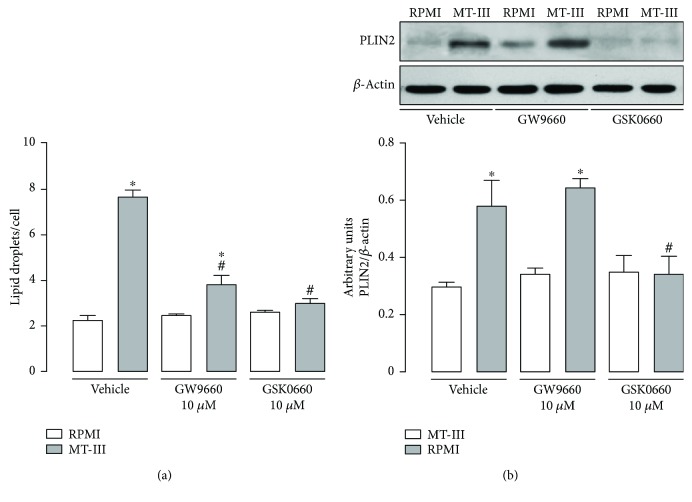
Both PPAR-*γ* and PPAR-*β*/*δ* are involved in MT-III-induced LD formation in macrophages, but only PPAR-*β/δ* is required for MT-III-induced PLIN2 protein expression in these cells. Peritoneal macrophages were incubated with GW9662 or GSK0660 (10 *μ*M) compounds for 1 h and then with MT-III (0.4 *μ*M) for 12 h. (a) LDs were quantified using light microscopy after osmium staining. Each bar represents the mean ± SEM LDs/cell in 50 counted cells. Values represent means ± SEM from 3–5 animals. ^∗^*p* < 0.05 compared with control group; ^#^*p* < 0.05 compared with vehicle-treated MT-III-stimulated cells. (b) Western blotting and densitometric analysis of the band intensities of PLIN2 and *β*-actin (loading control) in macrophage extracts. The densities (in arbitrary units) were normalized with those of *β*-actin. Results are expressed as mean ± SEM from three experiments. ^∗^*p* < 0.05 compared with controls. ^#^*p* < 0.05 compared with vehicle-treated MT-III-stimulated cells.

Participation of PPAR-*γ* and PPAR-*β*/*δ* in the signaling triggered by fatty acids has been previously reported [19]. Therefore, the involvement of these factors in MT-III-induced LD biogenesis was investigated using pharmacological approaches. The effects of selected pharmacological treatments were evaluated 12 h after incubation of macrophages with MT-III. As seen in Figure 1(a), pretreatment of cells with GW9662 (10 *μ*M), a PPAR-*γ* antagonist, caused 74% reduction in the number of LDs in MT-III-stimulated macrophages when compared with vehicle-treated macrophages stimulated with MT-III. However, pretreatment of cells with the PPAR-*β*/*δ* antagonist, GSK0660 abolished LD formation induced by MT-III in macrophages when compared with vehicle-treated macrophages stimulated with MT-III. In an attempt to better understand the involvement of PPAR-*γ* and PPAR-*β*/*δ* in MT-III-induced LD formation, we further evaluated the contribution of these transcription factors in PLIN2 protein expression induced by MT-III. PLIN2 is a structural protein implicated in LD assembly [5, 6] and transcriptionally controlled by factors involved in lipid homeostasis, including PPARs [11].  Figure 1(b) shows that PLIN2 protein expression was abolished in cells pretreated with the antagonist of PPAR-*β*/*δ* GSK0660 and then stimulated with MT-III. However, the treatment of cells with GW9662 followed by incubation with MT-III did not alter PLIN2 protein expression induced by this sPLA_2_. Altogether, these data indicate that both PPAR-*γ* and PPAR-*β/δ* are involved in LD formation stimulated by MT-III in macrophages while only PPAR-*β*/*δ* acts by increasing PLIN2 protein expression.

### 3.2. MT-III Upregulates PPAR-*γ* and PPAR-*β/δ* Protein Expression in Macrophages

**Figure 2:**
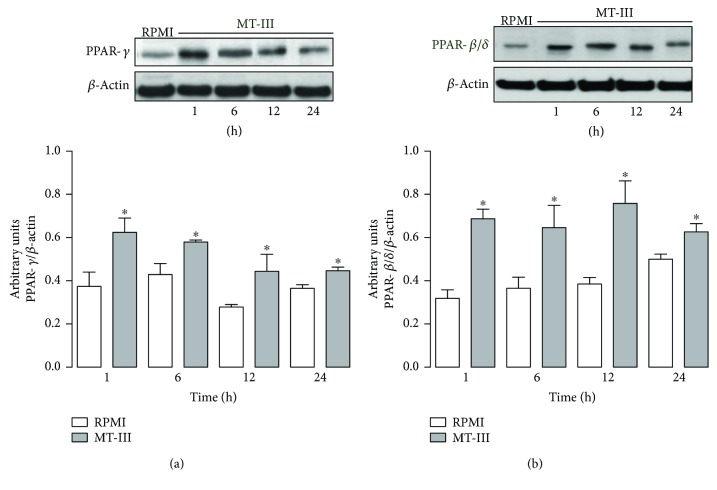
MT-III induces upregulation of PPAR-*γ* and PPAR-*β*/*δ* protein expression in macrophages. Peritoneal macrophages were incubated with MT-III (0.4 *μ*M) or RPMI (control) for 1, 6, 12, and 24 h. (a, b) Western blotting and densitometric analysis of the band intensities of immunoreactive PPAR-*γ* (a) or PPAR-*β*/*δ* (b) and *β*-actin (loading control) in macrophage extracts. Results are expressed as mean ± SEM from three experiments. ^∗^*p* < 0.05 compared with controls.

As an additional mechanism involved in lipid accumulation induced by MT-III in peritoneal macrophages, we investigated the ability of MT-III to upregulate PPAR-*γ* and PPAR-*β*/*δ* protein expression. Levels of PPAR-*γ* and PPAR-*β/δ* protein expression were analyzed by Western blot in cells stimulated with MT-III or RPMI (control) for selected time intervals (1–24 h). As seen in Figure 2(a), levels of PPAR-*γ* protein expression were increased in macrophages stimulated by MT-III in all time windows evaluated (1–24 h) when compared with the corresponding controls. Similarly, Figure 2(b) shows that MT-III increased PPAR-*β/δ* protein expression in macrophages from 1 up to 24 h, compared to control cells. Taken together, these data demonstrate the capacity of MT-III to induce PPAR*γ* and PPAR-*β/δ* protein expression in peritoneal macrophages.

### 3.3. MT-III Induces 15-d-PGJ_2_ Production via COX-1 Pathway and Activates PPAR-*γ* and PPAR-*β/δ*

**Figure 3:**
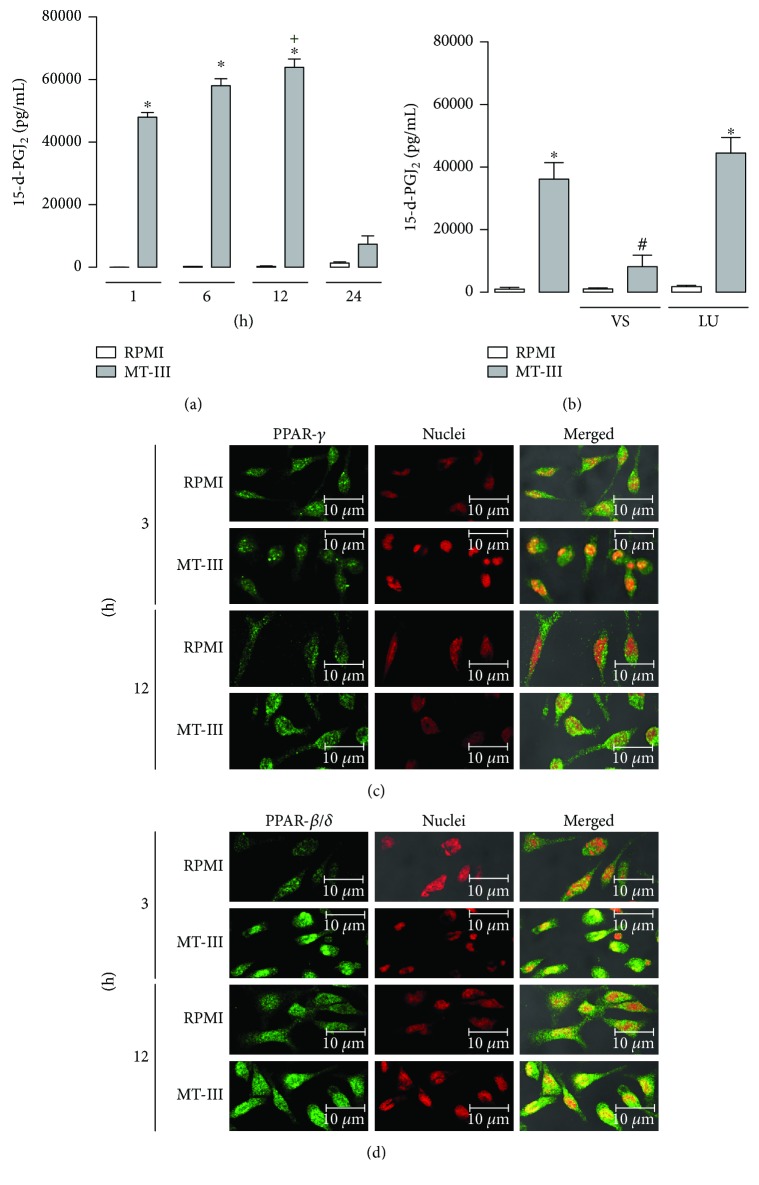
MT-III induces 15-d-PGJ_2_ production dependent on COX-1 and COX-2. PPAR-*γ* and PPAR-*β*/*δ* are activated by MT-III. (a) Quantification of 15-d-PGJ_2_ in macrophage culture supernatants by specific EIA. ^∗^*p* < 0.05 compared with control group; ^+^*p* < 0.05 as compared with MT-III-stimulated cells for 1 h. (b) Macrophages were pretreated with COX-1 (valeryl salicylate (VS)), 10 *μ*M, or COX-2 (lumiracoxib (LU)) 10 *μ*M inhibitors for 1 h before stimulation with MT-III (0.4 *μ*M) for 6 h, and 15-d-PGJ_2_ production was quantified by ELISA. ^∗^*p* < 0.05 compared with control group; ^#^*p* < 0.05 compared with MT-III- stimulated cells. (c, d) Macrophages incubated with RPMI (control) or MT-III (0.4 *μ*M) for 3 or 12 h were labelled with anti-PPAR-*γ* (b) or PPAR-*β*/*δ* (d) conjugated to Alexa 488 (green). Nuclei were stained with iodide propidium. The pictures are representative of three independent experiments.

It is known that lipid mediators, mainly 15-d-PGJ_2_, are potent endogenous activators of PPAR family [20, 21]. We then evaluated the ability of MT-III to induce 15-d-PGJ_2_ production in peritoneal macrophages. 15-d-PGJ_2_ levels were measured in supernatants from macrophages incubated with MT-III (0.4 *μ*M) or RPMI only (control). As shown in Figure 3(a), incubation of macrophages with MT-III induced a significant increase in 15-d-PGJ_2_ production between 1 and 12 h. Maximal 15-d-PGJ_2_ production was observed after 12 h of incubation with MT-III. These results reveal the capacity of MT-III to induce 15-d-PGJ_2_ production in macrophages. Pretreatment of cells with COX-1 inhibitor valeryl salicylate significantly reduced 15-d-PGJ_2_ production while pretreatment with COX-2 inhibitor, lumiracoxib, did not affect 15-d-PGJ_2_ production induced by MT-III (Figure 3(b)). Therefore, 15-d-PGJ_2_ production is dependent on COX-1, but not COX-2 pathway in macrophages stimulated by MT-III.

To confirm the activation of these factors by MT-III, an immunofluorescence assay was performed to evaluate translocation of PPAR-*γ* and PPAR-*β*/*δ* into the nucleus of macrophages. As illustrated in Figure 3(c), macrophages stimulated with RPMI (control) for 3 or 12 h exhibited a fluorescent staining (green) for PPAR-*γ*, which did not colocalize to nucleus staining (red). In macrophages stimulated with MT-III (0.4 *μ*M) for 3 h, fluorescent labeling for PPAR-*γ* was seen colocalized in the nucleus, indicating translocation of this factor to the nuclear region.  Figure 3(d) illustrates control macrophages incubated with RPMI (control) and stained for PPAR-*β*/*δ*. In this condition, cells presented a fluorescent staining for PPAR-*β/δ*, with the absence of colocalization with nucleus staining  after 3 and 12 h of incubation. However, incubation of cells with MT-III (0.4 *μ*M) induced a strong staining for this factor, which was colocalized with nucleus at 3 or 12 h of incubation, evidencing translocation of this factor into cell nucleus.

### 3.4. MT-III-Induced LD Formation Is a Platform to 15-d-PGJ_2_ Production

**Figure 4:**
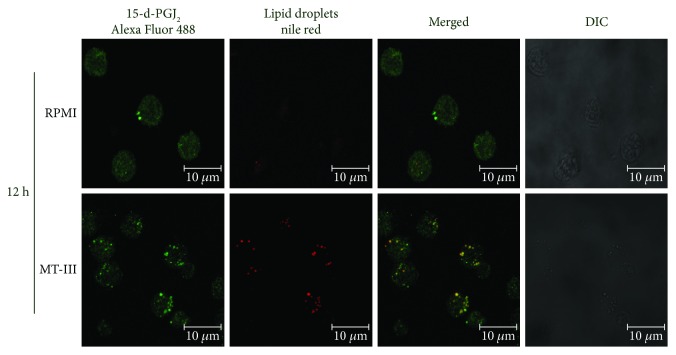
MT-III-induced LDs compartmentalize 15-d-PGJ_2_ in peritoneal macrophages. Macrophages incubated with RPMI (control) or MT-III (0.4 *μ*M) for 12 h were labeled for LDs (Nile Red) and for 15-d-PGJ_2_ (anti-15-d-PGJ_2_ antibody, Enzo Life Sciences). The merged image shows colocalization of 15-d-PGJ_2_ to LDs. The pictures are representative of three independent experiments.

LDs are lipid inclusions implicated in prostanoid biosynthesis [10]. Considering the increased levels of 15-d-PGJ_2_ in MT-III-stimulated macrophages, we hypothesize that LDs could constitute a relevant site for production of 15-d-PGJ_2_. In order to investigate this hypothesis, macrophages stimulated with MT-III or RPMI (control) were incubated with EDAC to immobilize the newly synthesized eicosanoids [18] and immunostained with antibodies against 15-d-PGJ_2_. LDs were stained with Nile Red. As illustrated in Figure 4, macrophages stimulated with MT-III (0.4 *μ*M) for 12 h exhibited a (green) cytoplasmic staining pattern for 15-d-PGJ_2_. This same pattern was not observed in the unstimulated control cells, which exhibited diffuse staining. Fluorescent Nile Red-labeled LBs were also visualized 12 h after stimulation by MT-III and were absent in unstimulated control macrophages. Overlapping images show that stained cytoplasmic 15-d-PGJ_2_ matched perfectly with neutral lipid inclusions in MT-III-stimulated macrophages indicating that 15-d-PGJ_2_ colocalizes to LDs. This demonstrates that MT-III-induced LDs constitute cytoplasmic platforms involved in 15-d-PGJ_2_ production.

### 3.5. Neutral Lipid Biosynthesis Is Relevant to MT-III-Induced LD Formation

**Figure 5:**
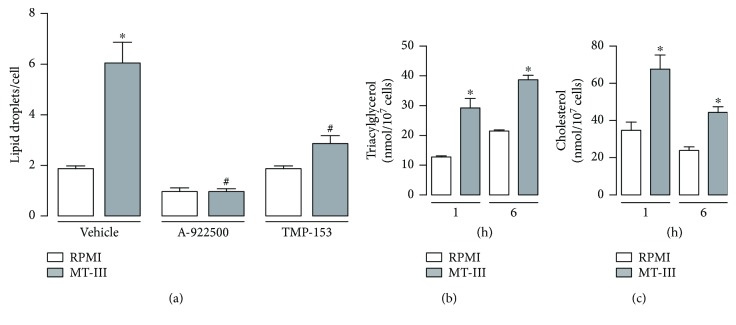
DGAT and ACAT enzymes are essential for LD formation induced by MT-III in macrophages. (a) Peritoneal macrophages were incubated with A922500 (100 nM), a DGAT inhibitor or TMP-153 (100 nM), an ACAT inhibitor or vehicle (<1% DMSO/RPMI) for 1 h and then with MT-III (0.4 *μ*M) for 12 h. LDs were quantified using light microscopy after osmium staining. Each bar represents the mean ± SEM LDs/cell in 50 counted cells. ^∗^*p* < 0.05 compared with control group; ^#^*p* < 0.05 compared with vehicle-treated MT-III-stimulated cells. Levels of (b) TAG and (c) CE in 1.10^7^ macrophages extracts incubated with MT-III (0.4 *μ*M) or RPMI (control) for 1 and 6 h quantified by specific detection kits. Values represent means ± SEM from 3–5 animals. ^∗^*p* < 0.05 compared with the control group.

It has been demonstrated that activation of PPARs triggers an increase in enzymatic activity of enzymes involved in neutral lipid synthesis [22]. DGAT and ACAT are the main enzymes responsible for the synthesis of triacylglycerol (TAG) and cholesterol (CE), respectively, both present in LD core [23, 24]. Our results show that pretreatment of cells with A922500, a DGAT inhibitor, or TMP-153, an ACAT inhibitor, abolished LD formation induced by MT-III when compared with vehicle-treated cells stimulated with MT-III (Figure 5(a)). As shown in Figures 5(b) and 5(c), incubation of macrophages with MT-III induced a significant increase in levels of TAG and CE, respectively, between 1 and 6 h in comparison with respective control cells. These results indicate the involvement of DGAT and ACAT in MT-III-induced LD formation and the composition of these lipid organelles.

### 3.6. CD36 Contributes to LD Formation and COX-2 Protein Expression Induced by MT-III

**Figure 6:**
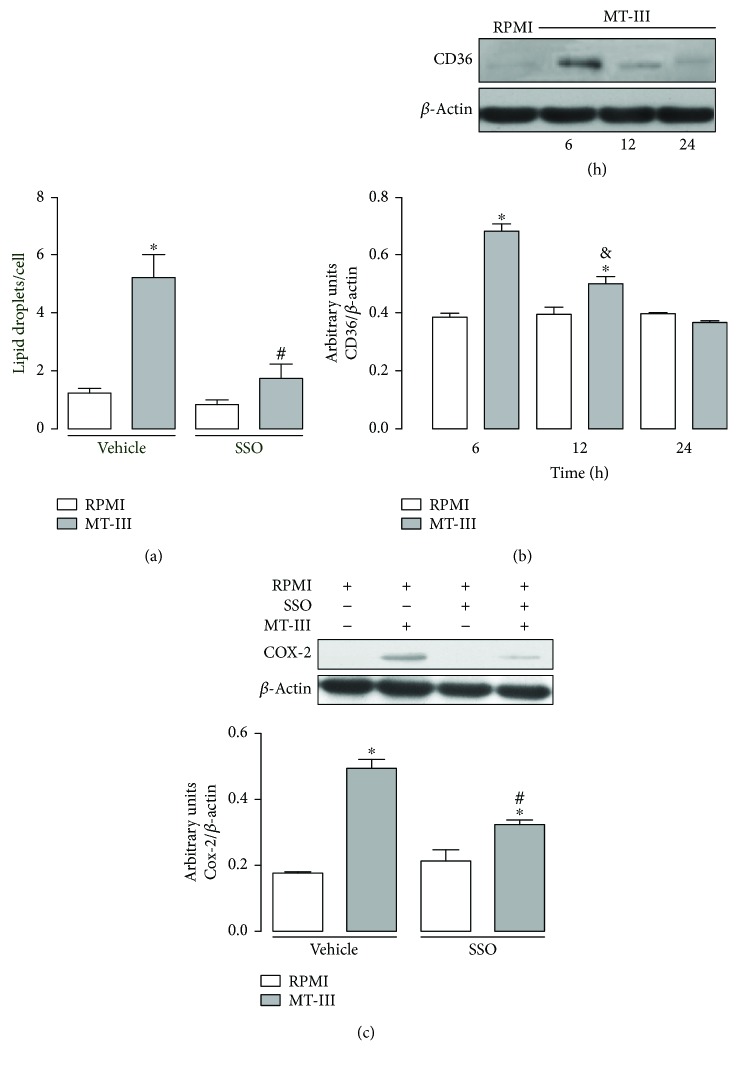
MT-III-induced LD formation and COX-2 protein expression in macrophages are dependent on CD36. (a) Peritoneal macrophages were incubated with SSO (25 *μ*M) compound or vehicle (<1% DMSO/RPMI) for 30 min and then with MT-III (0.4 *μ*M) for 12 h. LDs were quantified using light microscopy after osmium staining. Each bar represents the mean ± SEM LDs/cell in 50 counted cells. Values represent means ± SEM from 3–5 animals. ^∗^*p* < 0.05 compared with the control group; ^#^*p* < 0.05 compared with vehicle-treated MT-III-stimulated cells. (b) Western blotting and densitometric analysis of CD36 and *β*-actin (loading control) in macrophage extracts. Peritoneal macrophages were incubated with MT-III (0.4 *μ*M) or RPMI (control) for 6, 12, and 24 h. ^∗^*p* < 0.05 compared with controls; ^&^*p* < 0.05 compared with MT-III 6 h. (c) Western blotting and densitometric analysis of COX-2 and *β*-actin (loading control) in macrophage extracts pretreated with CD36 antagonist, SSO. ^#^*p* < 0.05 compared with vehicle-treated MT-III- stimulated cells. Peritoneal macrophages were incubated with SSO (25 *μ*M) compound or vehicle (<1% DMSO/RPMI) for 30 min and then with MT-III (0.4 *μ*M) for 12 h. The densities (in arbitrary units) were normalized with those of *β*-actin. Results are expressed as mean ± SEM from three experiments. ^∗^*p* < 0.05 compared with controls.

Macrophages internalize lipids by different scavenger receptors, among which is CD36. This receptor binds oxidized phospholipids/lipoproteins and long-chain fatty acids, leading to LD formation and, as a consequence, to differentiation of macrophages to foam cells during atherosclerotic processes [25, 26]. To verify the role of CD36 in LD formation induced by MT-III, we evaluated the effect of sulfosuccinimidyl oleate compound (SSO), a CD36 antagonist, on MT-III-induced LD formation. As shown in Figure 6(a), pretreatment of macrophages with SSO (25 *μ*M) abolished LD formation induced by MT-III in comparison with vehicle-treated cells, stimulated with MT-III. This result indicates that CD36 is relevant to MT-III-induced LD formation. Considering the relevance of CD36 to intracellular lipid accumulation in macrophages, we next investigated whether MT-III would increase the levels of CD36 in macrophages. For this purpose, Western blotting analysis was performed, in cells incubated and not incubated with MT-III or medium (control) for 6, 12, and 24 h. This analysis revealed a significant increase of CD36 protein levels in cells stimulated with MT-III from 6 up to 12 h, with a maximum level seen at 6 hours of incubation, in comparison with the respective control (Figure 6(b)). Taken together, these data show that CD36 scavenger receptor is an important factor involved in LD formation in cells stimulated by MT-III. Therefore, upregulation of CD36 expression can be an additional mechanism implicated in lipid accumulation induced by MT-III in macrophages. Considering CD36 a superficial membrane receptor, we investigate the contribution of this receptor in the activation of COX-2 pathway in macrophages stimulated by MT-III. As shown in Figure 6(c), pretreatment of macrophages with SSO (25 *μ*M) reduced the protein expression of COX-2 induced by MT-III in comparison with vehicle-treated cells and stimulated with MT-III. This result indicates that CD36 is relevant to MT-III-induced COX-2 protein expression.

### 3.7. Upregulation of CD36 Protein Expression Is Regulated by PPAR-*γ* and PPAR-*β*/*δ* Activation in Macrophages Stimulated by MT-III

**Figure 7:**
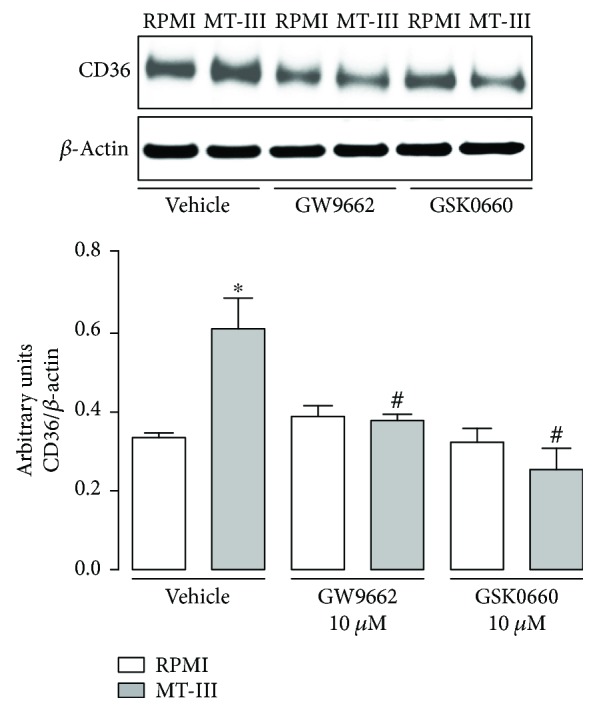
MT-III-induced CD36 protein expression is dependent on PPAR-*γ* and PPAR-*β*/*δ* activation. Peritoneal macrophages were incubated with GW9662 (10 *μ*M) or GSK0660 (10 *μ*M) compounds or vehicle (<1% DMSO/RPMI) for 1 h and then with MT-III (0.4 *μ*M) for 6 h. Western blotting and densitometric analysis of CD36 receptor and *β*-actin (loading control) in macrophage extracts. The densities (in arbitrary units) were normalized with those of *β*-actin. Results are expressed as mean ± SEM from three experiments. ^∗^*p* < 0.05 compared with controls; ^#^*p* < 0.05 compared with vehicle-treated MT-III-stimulated cells.

Considering the relevance of CD36 receptor to metabolism and accumulation of lipids in macrophages and previous reports demonstrating that this receptor is under transcriptional control of PPAR family [27], we evaluated the participation of PPAR-*γ* and PPAR-*β*/*δ* in the upregulation of CD36 expression induced by MT-III. Pretreatment of cells with either GW9662 (PPAR-*γ* antagonist) or GSK0660 (PPAR-*β*/*δ* antagonist) abolished the increase of CD36 protein expression caused by MT-III in comparison with vehicle-treated cells stimulated with MT-III (Figure 7). These data indicate that the increase of levels of CD36 protein expression induced by MT-III in macrophages is dependent on PPAR-*γ* and PPAR-*β/δ* activation.

## 4. Discussion

Lipid accumulation in monocytes and macrophages is responsible for differentiation of these cells into foam cells and represents a key step in the development of atherosclerosis [5, 28]. Therefore, these cells have been used as targets to understand the inductors and mechanisms involved in atherosclerotic process. Hereby, we demonstrated the activation, expression, and contribution of different factors involved in lipid accumulation induced by MT-III, a group IIA sPLA_2_, in phagocytic cells. Our results indicate that MT-III is able to induce activation of PPAR-*γ* and PPAR-*β*/*δ* in macrophages, since both transcription factors were translocated into nucleus in cells stimulated by this sPLA_2_. In the present experimental conditions, translocation of PPAR-*β*/*δ* into the nucleus was higher than that of PPAR-*γ* and showed a long-term profile. Considering that PPARs are activated by lipid mediators and free fatty acids [29] and that arachidonic acid (AA) was shown to activate PPAR-*γ* in alveolar macrophages [30], the ability of MT-III to induce release of AA and other polyunsatured fatty acids from macrophage membranes [17, 31] may provide a favorable environment for activation of these factors. Although, activation of PPAR-*γ* and PPAR-*β*/*δ* by LDL modified by a sPLA_2_ from *Naja mossambica* snake venom has been reported in the literature [32]; to our knowledge, this is the first demonstration that a group IIA sPLA_2_ per se induces activation of PPAR-*γ* and PPAR-*β/δ*. The involvement of PPAR-*γ* and PPAR-*β/δ* in LD formation and regulation of expression of PLIN2, a protein involved in the uptake of fatty acids and differentiation of macrophages into foam cells, have been demonstrated in the literature [33–35]. Our findings that inhibition of PPAR-*β/δ* abolished both LD formation and PLIN2 protein expression induced by MT-III indicate that PPAR-*β/δ* by regulating PLIN2 expression plays a crucial role in LD formation induced by the studied sPLA_2_. On the other hand, while inhibition of PPAR-*γ* activation reduced LD formation, no modification of PLIN2 protein expression was observed, evidencing that upon stimulus by MT-III, PPAR-*γ* and PPAR-*β/δ* regulate LB formation by targeting different genes related to lipid uptake. This result contrasts with those demonstrating that PPAR-*γ* regulates PLIN2 protein expression induced by oxidized low-density lipoprotein or troglitazone, a PPAR-*γ* agonist [36, 37]. Furthermore, the data here obtained shows that MT-III is able to upregulate PPAR-*γ* and PPAR-*β/δ* protein expression, underscoring an additional mechanism by which this group IIA sPLA_2_ induces LD formation. In addition, our present results showed a marked and early production of 15-d-PGJ_2,_ which is dependent on COX-1, but not COX-2 pathway. These findings are in line with previous results showing that MT-III induces an early increase of COX-1 activation in macrophages [31]. On the other hand, upregulation of COX-2 protein expression observed in macrophages stimulated by MT-III may be related to biosynthesis of proinflammatory prostaglandins, which were not investigated in the present study, since inhibition of COX-2 did not affect MT-III-induced release of 15-d-PGJ_2_. This resolutive mediator is recognized as a relevant activator of PPAR family and has been found in macrophages of atherosclerotic lesions [38]. Therefore, the tight correlation between the profiles of 15-d-PGJ_2_ release and activation of PPARs seen in the present study implicate 15-d-PGJ_2_ as an important player in MT-III-induced activation of PPARs. Moreover, our findings that MT-III caused an increase of 15-d-PGJ_2_ intracellular pools colocalized to LDs in MT-III-stimulated macrophages evidence that these organelles are sites for production of 15-d-PGJ_2_. These data are in accordance with previous reports on the involvement of LDs in the production of inflammatory mediators acting as a platform for enhanced synthesis of lipid mediators. Taken together, these results give support to our contention that these organelles are important sites for 15-d-PGJ_2_ synthesis [39–42]. Although the mechanisms involved in formation of 15-d-PGJ_2_ upon MT-III stimulus were not presently investigated, we herein hypothesize that, by providing a highly hydrophobic environment, LDs are suitable sites for 15-d-PGJ_2_ production, since this mediator is formed through the spontaneous dehydration of PGD_2_ [38]. Additionally, our findings reinforce the idea that LDs can also be implicated in the negative regulation of inflammatory response, as 15-d-PGJ_2_ is a recognized preresolving mediator, signaling the switch from acute inflammatory phase to the resolving phase of inflammation [43, 44]. Hence, the formation of this mediator upon the action of MT-III may reflect a regulatory mechanism to dampen the inflammatory reaction elicited by the enzyme.

In addition, our present results demonstrate that LD formation induced by MT-III is largely dependent on the endoplasmic reticulum enzymes DGAT and ACAT, since inhibitors of their enzymatic activities abolished LD formation induced by MT-III. Moreover, the increased levels of TAG and CE detected under MT-III stimulation give support to the participation of DGAT and ACAT in LD formation induced by this sPLA_2_. Our data are in agreement with findings that DGAT and ACAT are involved in LD formation in a number of inflammatory conditions [45–47]. PPAR-*γ* agonists have been described to induce expression of DGAT and the consequent increase of triacylglycerol storages [48]. However, gene expression of DGAT and ACAT was not induced by MT-III in the present experimental condition (data not shown)

Another PPAR-regulated factor relevant for LD formation in macrophages during atherogenic processes is the receptor CD36 [49]. Our results demonstrating that inhibition of CD36 markedly reduced LD formation imply the involvement of this scavenger receptor in foam cell formation induced by MT-III in macrophages. In support of this finding, upregulation of CD36 protein expression by this sPLA_2_ was observed. Moreover, our data showed that the increased expression of this receptor induced by MT-III is regulated by PPAR-*β*/*δ* and PPAR-*γ*, since inhibition of these transcription factors abolished CD36 protein expression. It is known that activation of the CD36 receptor triggers a signaling pathway that leads to the synthesis of inflammatory lipid mediators [27, 50]. Furthermore, the observation that inhibition of CD36 downregulated COX-2 protein expression suggests a role for this receptor in the formation of prostanoids in the late periods of activation of macrophages induced by MT-III. Altogether, these findings emphasize the crucial role of PPAR-*β/δ* and PPAR-*γ* for lipid accumulation in macrophages stimulated by group IIA-sPLA_2_.

**Figure 8:**
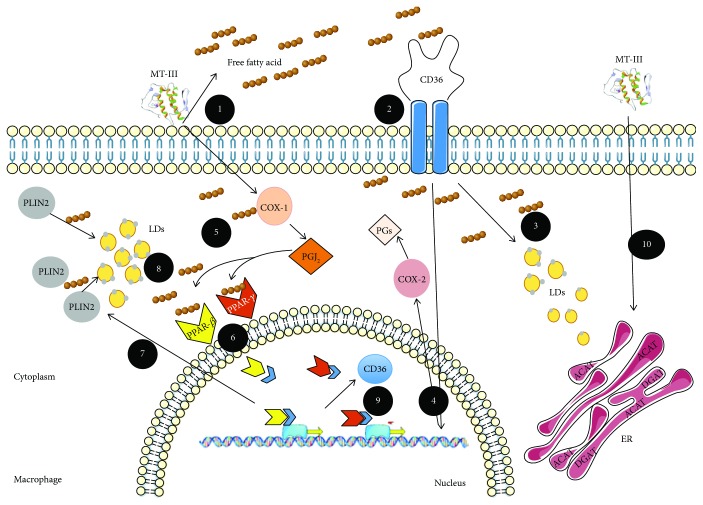
Schematic illustration of the mechanisms involved in foam cell formation induced by MT-III a GIIA snake venom sPLA_2_. (1) MT-III by acting on cellular membrane of macrophages generates free fatty acids, which will be internalized by CD36 scavenger receptors; (2) activation of C36 receptor leads to formation of (3) lipid droplets and (4) expression of COX-2. (5) MT-III induces production of 15-d-PGJ_2_ via COX-1 pathway (6). This mediator and free fatty acids activate the cytoplasmic transcription factors PPARs; (7) PLIN2, scaffold protein for lipid droplet assembly, is under transcriptional control of PPAR-*β/δ* and favors the increase of (8) lipid droplet formation. (9) Both PPAR-*γ* and PPAR-*β/δ* upregulate CD36 protein expression forming a positive loop for generation of lipid droplets in macrophages. (10) MT-III also activates endoplasmic reticulum enzymes (DGAT and ACAT) crucial for formation of lipid droplets.

In conclusion, our data highlight the mechanisms involved in LD formation induced by MT-III, a secreted phospholipase A_2_ from snake venom (Figure 8). MT-III was able to increase the expression of the transcription factors PPAR-*γ*, PPAR-*β/δ*, and of the scavenger receptor CD36 in peritoneal macrophages. Also, MT-III activated these nuclear transcription factors. Inhibition of these factors as well as of CD36 and ACAT and DGAT enzymes also reduced LD formation induced by MT-III, thus implicating these factors in LDs formation induced by PLA_2_ MT-III. PPAR-*β/δ*, but not PPAR-*γ*, by inducing PLIN2 expression, plays a key role in LD formation induced by MT-III. Both transcription factors contribute to MT-III-induced CD36 protein expression. CD36 in turn regulated MT-III-induced COX-2 protein expression. MT-III also induced formation and release of 15-d-PGJ_2_, an activator of PPARs in macrophages. Therefore, LDs constitute a site for production of this lipid mediator upon MT-III-induced stimulation. Considering the homology between mammalian-secreted phospholipase A_2_ and phospholipase A_2_ found in snake venoms, our results may contribute to the understanding of the actions and mechanisms triggered by this class of enzymes related to lipid accumulation in phagocytic cells in a variety of inflammatory and pathological conditions.

## Additional Points

*Summary Statement*. Lipid droplet formation induced by a snake venom GIIA sPLA_2_ in macrophages depends on lipid metabolism factors mainly PPAR-*γ* and PPAR-*β*/*δ*, providing new insights into mechanisms of macrophage foam cell formation.

## References

[1] Lambeau G., Gelb M. H. (2008). Biochemistry and physiology of mammalian secreted phospholipases A2. *Annual Review of Biochemistry*.

[2] Murakami M., Taketomi Y., Miki Y., Sato H., Yamamoto K., Lambeau G. (2014). Emerging roles of secreted phospholipase A_2_ enzymes: the 3rd edition. *Biochimie*.

[3] Six D. A., Dennis E. A. (2000). The expanding superfamily of phospholipase A_2_ enzymes: classification and characterization. *Biochimica et Biophysica Acta (BBA) - Molecular and Cell Biology of Lipids*.

[4] Tonello F., Rigoni M., Gopalakrishnakone P., Inagaki H., Mukherjee A., Rahmy T., Vogel C. W. (2015). Cellular mechanisms of action of snake phospholipase A2 toxins. *Snake Venoms*.

[5] Paul A., Chang B. H. J., Li L., Yechoor V. K., Chan L. (2008). Deficiency of adipose differentiation-related protein impairs foam cell formation and protects against atherosclerosis. *Circulation Research*.

[6] Becker L., Gharib S. A., Irwin A. D. (2010). A macrophage sterol-responsive network linked to atherogenesis. *Cell Metabolism*.

[7] Plakkal Ayyappan J., Paul A., Goo Y. H. (2016). Lipid droplet-associated proteins in atherosclerosis (review). *Molecular Medicine Reports*.

[8] Onal G., Kutlu O., Gozuacik D., Dokmeci Emre S. (2017). Lipid droplets in health and disease. *Lipids in Health and Disease*.

[9] Welte M. A., Gould A. P. (2017). Lipid droplet functions beyond energy storage. *Biochimica et Biophysica Acta (BBA) - Molecular and Cell Biology of Lipids*.

[10] Araújo-Santos T., Prates D. B., Andrade B. B. (2010). *Lutzomyia longipalpis* saliva triggers lipid body formation and prostaglandin E_2_ production in murine macrophages. *PLoS Neglected Tropical Diseases*.

[11] Nakata A., Nakagawa Y., Nishida M. (1999). CD36, a novel receptor for oxidized low-density lipoproteins, is highly expressed on lipid-laden macrophages in human atherosclerotic aorta. *Arteriosclerosis, Thrombosis, and Vascular Biology*.

[12] Febbraio M., Podrez E. A., Smith J. D. (2000). Targeted disruption of the class B scavenger receptor CD36 protects against atherosclerotic lesion development in mice. *The Journal of Clinical Investigation*.

[13] Rader D. J., Puré E. (2005). Lipoproteins, macrophage function, and atherosclerosis: beyond the foam cell?. *Cell Metabolism*.

[14] Walther T. C., Farese R. V. (2012). Lipid droplets and cellular lipid metabolism. *Annual Review of Biochemistry*.

[15] Gutierrez J. M., Lomonte B. (1995). Phospholipase A_2_ myotoxins from *Bothrops* snake venoms. *Toxicon*.

[16] Takayama K., Mitchell D. H., Din Z. Z., Mukerjee P., Li C., Coleman D. L. (1994). Monomeric re lipopolysaccharide from Escherichia coli is more active than the aggregated form in the Limulus amebocyte lysate assay and in inducing Egr-1 mRNA in murine peritoneal macrophages. *Journal of Biological Chemistry*.

[17] Leiguez E., Giannotti K. C., Moreira V. (2014). Critical role of TLR2 and MyD88 for functional response of macrophages to a *Group* IIA-secreted phospholipase A_2_ from snake venom. *PLoS One*.

[18] Bandeira-Melo C., Weller P. F., Bozza P. T., Chiarini-Garcia H., Melo R. (2011). EicosaCell – an immunofluorescent-based assay to localize newly synthesized eicosanoid lipid mediators at intracellular sites. *Light Microscopy*.

[19] Varga T., Czimmerer Z., Nagy L. (2011). PPARs are a unique set of fatty acid regulated transcription factors controlling both lipid metabolism and inflammation. *Biochimica et Biophysica Acta (BBA) - Molecular Basis of Disease*.

[20] Forman B. M., Tontonoz P., Chen J., Brun R. P., Spiegelman B. M., Evans R. M. (1995). 15-deoxy-∆^12,14^-prostagalandin J_2_ is a ligand for the adipocyte determination factor PPAR*γ*. *Cell*.

[21] Kliewer S. A., Lenhard J. M., Willson T. M., Patel I., Morris D. C., Lehmann J. M. (1995). A prostaglandin J_2_ metabolite binds peroxisome proliferator-activated receptor *γ* and promotes adipocyte differentiation. *Cell*.

[22] Blanchard P. G., Turcotte V., Côté M. (2016). Peroxisome proliferator-activated receptor *γ* activation favours selective subcutaneous lipid deposition by coordinately regulating lipoprotein lipase modulators, fatty acid transporters and lipogenic enzymes. *Acta Physiologica*.

[23] Khelef N., Buton X., Beatini N. (1998). Immunolocalization of acyl-coenzyme A: cholesterol *O*-acyltransferase in macrophages. *Journal of Biological Chemistry*.

[24] Stone S. J., Myers H. M., Watkins S. M. (2004). Lipopenia and skin barrier abnormalities in DGAT2-deficient mice. *Journal of Biological Chemistry*.

[25] Huh H. Y., Pearce S. F., Yesner L. M., Schindler J. L., Silverstein R. L. (1996). Regulated expression of CD36 during monocyte-to-macrophage differentiation: potential role of CD36 in foam cell formation. *Blood*.

[26] Park Y. M. (2014). CD36, a scavenger receptor implicated in atherosclerosis. *Experimental & Molecular Medicine*.

[27] Bujold K., Rhainds D., Jossart C., Febbraio M., Marleau S., Ong H. (2009). CD36-mediated cholesterol efflux is associated with PPAR*γ* activation via a MAPK-dependent COX-2 pathway in macrophages. *Cardiovascular Research*.

[28] Libby P., DiCarli M., Weissleder R. (2010). The vascular biology of atherosclerosis and imaging targets. *The Journal of Nuclear Medicine*.

[29] Desvergne B., Wahli W. (1999). Peroxisome proliferator-activated receptors: nuclear control of metabolism. *Endocrine Reviews*.

[30] Alaoui-El-Azher M., Wu Y., Havet N. (2002). Arachidonic acid differentially affects basal and lipopolysaccharide-induced sPLA_2_-IIA expression in alveolar macrophages through NF-*κ*B and PPAR-*γ*-dependent pathways. *Molecular Pharmacology*.

[31] Moreira V., Gutiérrez J. M., Soares A. M., Zamunér S. R., Purgatto E., Teixeira C. . F. P. (2008). Secretory phospholipases A_2_ isolated from *Bothrops asper* and from *Crotalus durissus terrificus* snake venoms induce distinct mechanisms for biosynthesis of prostaglandins E_2_ and D_2_ and expression of cyclooxygenases. *Toxicon*.

[32] Namgaladze D., Morbitzer D., von Knethen A., Brune B. (2010). Phospholipase A_2_–modified low-density lipoprotein activates macrophage peroxisome proliferator–activated receptors. *Arteriosclerosis, Thrombosis, and Vascular Biology*.

[33] Vosper H., Patel L., Graham T. L. (2001). The peroxisome proliferator-activated receptor *δ* promotes lipid accumulation in human macrophages. *Journal of Biological Chemistry*.

[34] Schadinger S. E., Bucher N. L., Schreiber B. M., Farmer S. R. (2005). PPAR*γ*2 regulates lipogenesis and lipid accumulation in steatotic hepatocytes. *American Journal of Physiology Endocrinology and Metabolism*.

[35] Almeida P. E., Silva A. R., Maya-Monteiro C. M. (2009). *Mycobacterium bovis* bacillus Calmette-Guérin infection induces TLR2-dependent peroxisome proliferator-activated receptor *γ* expression and activation: functions in inflammation, lipid metabolism, and pathogenesis. *The Journal of Immunology*.

[36] Motomura W., Inoue M., Ohtake T. (2006). Up-regulation of ADRP in fatty liver in human and liver steatosis in mice fed with high fat diet. *Biochemical and Biophysical Research Communications*.

[37] Liu Q., Dai Z., Liu Z. (2010). Oxidized low-density lipoprotein activates adipophilin through ERK1/2 signal pathway in RAW264.7 cells. *Acta Biochimica et Biophysica Sinica*.

[38] Shibata T., Kondo M., Osawa T., Shibata N., Kobayashi M., Uchida K. (2002). 15-deoxy-∆^12,14^-prostaglandin J_2_ a prostaglandin D_2_ metabolite generated during inflammatory processes. *Journal of Biological Chemistry*.

[39] Accioly M. T., Pacheco P., Maya-Monteiro C. M. (2008). Lipid bodies are reservoirs of cyclooxygenase-2 and sites of prostaglandin-E_2_ synthesis in colon cancer cells. *Cancer Cells*.

[40] D'Avila H., Roque N. R., Cardoso R. M., Castro-Faria-Neto H. C., Melo R. C. N., Bozza P. T. (2008). Neutrophils recruited to the site of *Mycobacterium bovis* BCG infection undergo apoptosis and modulate lipid body biogenesis and prostaglandin E_2_ production by macrophages. *Cellular Microbiology*.

[41] Bozza P. T., Magalhães K. G., Weller P. F. (2009). Leukocyte lipid bodies — biogenesis and functions in inflammation. *Biochimica et Biophysica Acta (BBA) - Molecular and Cell Biology of Lipids*.

[42] Giannotti K. C., Leiguez E., Carvalho A. E. Z. (2017). A snake venom group IIA PLA_2_ with immunomodulatory activity induces formation of lipid droplets containing 15-d-PGJ_2_ in macrophages. *Scientific Reports*.

[43] Scher J. U., Pillinger M. H. (2005). 15d-PGJ_2_: the anti-inflammatory prostaglandin?. *Clinical Immunology*.

[44] Hilliard M., Frohnert C., Spillner C. (2010). The anti-inflammatory prostaglandin 15-deoxy-Δ^12,14^-PGJ_2_ inhibits CRM1-dependent nuclear protein export. *Journal of Biological Chemistry*.

[45] Quittnat F., Nishikawa Y., Stedman T. T. (2004). On the biogenesis of lipid bodies in ancient eukaryotes: synthesis of triacylglycerols by a *toxoplasma* DGAT1-related enzyme. *Molecular and Biochemical Parasitology*.

[46] Matsuda D., Ohshiro T., Ohba M. (2009). The molecular target of rubimaillin in the inhibition of lipid droplet accumulation in macrophages. *Biological and Pharmaceutical Bulletin*.

[47] McFie P. J., Banman S. L., Kary S., Stone S. J. (2011). Murine diacylglycerol acyltransferase-2 (DGAT2) can catalyze triacylglycerol synthesis and promote lipid droplet formation independent of its localization to the endoplasmic reticulum. *Journal of Biological Chemistry*.

[48] Koliwad S. K., Streeper R. S., Monetti M. (2010). DGAT1-dependent triacylglycerol storage by macrophages protects mice from diet-induced insulin resistance and inflammation. *The Journal of Clinical Investigation*.

[49] Lee T. S., Lin C. Y., Tsai J. Y. (2009). Resistin increases lipid accumulation by affecting class A scavenger receptor, CD36 and ATP-binding cassette transporter-A1 in macrophages. *Life Sciences*.

[50] Kuda O., Jenkins C. M., Skinner J. R. (2011). CD36 protein is involved in store-operated calcium flux, phospholipase A2 activation, and production of prostaglandin E2. *Journal of Biological Chemistry*.

